# Comparison of Efficacy and Safety of Anti-Programmed Cell Death-1 Antibody Plus Lenvatinib and Chemotherapy as First-Line Therapy for Patients with Stage IV Gallbladder Cancer: A Real-World Study in a Chinese Population

**DOI:** 10.3390/biomedicines11112933

**Published:** 2023-10-30

**Authors:** Tiantian Wu, Changsheng Pu, Qiang Wang, Keming Zhang

**Affiliations:** Department of Hepatobiliary Surgery, Peking University International Hospital, No. 1, Life Garden Road, Zhongguancun Life Science Garden, Changping District, Beijing 102206, China; puchangsheng@pkuih.edu.cn (C.P.); wangqiang1@pkuih.edu.cn (Q.W.)

**Keywords:** anti-programmed cell death-1 antibody, targeted therapy, chemotherapy, gallbladder cancer

## Abstract

**Background:** The present study aimed to evaluate and compare the efficacy and safety of anti-programmed cell death protein 1 (anti-PD-1) antibody plus lenvatinib (tyrosine kinase inhibitor) therapy and chemotherapy as the first-line treatment to unresectable stage IV gallbladder cancer (GBC). **Methods:** We retrospectively analyzed the clinical data of patients with stage IV GBC who received chemotherapy or anti-PD-1 antibody combined with lenvatinib therapy at our hospital from March 2018 to October 2022. Patients with previous antitumor treatment were excluded. The overall survival (OS), progression-free survival (PFS), objective response rate (ORR), disease control rate (DCR), and adverse events (AEs) were assessed. **Results:** A total of 64 patients were enrolled, of which 33 patients received chemotherapy (gemcitabine + cisplatin) in the chemotherapy group, and 31 patients received anti-PD-1antibody (camrelizumab) combined with lenvatinib therapy in the combined therapy group. The median OS was 12.00 months in the combined therapy group and 10.00 months in the chemotherapy group (hazard ratio (HR), 0.57; 95% CI: 0.32–1.03; *p* < 0.05). The median PFS was 9.00 months in the combined therapy group and 6.00 months in the chemotherapy group (HR, 0.46; 95% CI: 0.25–0.84; *p* < 0.01). The ORR was 54.84% and 39.39% in the combined therapy and chemotherapy groups, respectively, and the difference was not significant (*p* = 0.22). The DCR was 80.65% and 72.72% in the combined therapy and chemotherapy groups, respectively (*p* = 0.46). One patient successfully underwent radical surgery after 8 months of combined therapy and achieved a pathological complete response. Furthermore, no patients experienced AEs of hematologic toxic effects in the combined therapy group compared with the chemotherapy group, demonstrating the advantage of the combined therapy. **Conclusions:** Anti-PD-1 antibody combined with lenvatinib may be a potentially effective and tolerable first-line treatment for unresectable stage IV GBC.

## 1. Introduction

Gallbladder cancer (GBC) is one of the most common primary malignant tumors of the biliary tract, with a worldwide occurrence of less than 2/100,000 individuals, and the geographic distribution of GBC incidence varies extensively [1]. In 2019, about 12,360 new cases of GBC and other biliary cancer were diagnosed in the United States; among those, 3960 patients died [2]. In China, 55,700 cases of GBC were diagnosed in 2016, with a crude incidence of 4.03 per 10^5^, and an age-standardized incidence rate by world standard population of 2.39 per 10^5^ [3]. Genetic and environmental factors are involved in the development of GBC, and the rate of female GBC is 2–3-times higher than that of male GBC worldwide [4,5]. Tobacco consumption, family history of gallstones, high concentrations of secondary bile acids, chemical exposure, and excessive fried food intake are the putative risk factors for GBC [6,7]. In China, gallstones are regarded as a major risk factor for GBC [8]. Among Chinese population, symptomatic gallstone disease was associated with increased risk of GBC, with an adjusted hazard ratio (HR) 3.70 (95% CI 2.88–4.87) [9]. The HRs were similar in men and women, with no heterogeneity by sex [9].

The prognosis of GBC is extremely poor; the median overall survival (OS) is approximately 4–7 months [10]. Radical surgery is probably the only curative treatment for GBC; however, most GBC cases are combined with advanced local infiltration or distant metastases. In China, most patients are diagnosed with stage III or IV GBC and have a limited opportunity for surgical resection. For unresectable cases, the conventionally recommended therapy is chemotherapy such as gemcitabine plus cisplatin [11,12]. Unfortunately, clinical outcomes are often poor owing to drug resistance or intolerance to side effects [13]. Recently, immune checkpoint inhibitors (ICIs) combined with tyrosine kinase inhibitors (TKIs) have achieved remarkable results in the treatment of a series of advanced malignant tumors, especially liver tumors [14,15,16,17]. Recent studies have demonstrated the safety and feasibility of this combined therapy or sequential therapy of targeted therapy/immunotherapy with systemic chemotherapy for advanced GBC [18,19,20]. Contrary to conventional systemic therapy, the clinical efficacy of chemo-free treatment using anti-programmed cell death protein 1 (anti-PD-1) antibodies plus a multi-target TKI agent lenvatinib has seldom been reported in unresectable GBC. Herein, we compared the clinical efficacy of chemo-free anti-PD-1 antibody plus lenvatinib and conventional chemotherapy as first-line treatment in patients with stage IV GBC. We believe that compared with conventional systemic chemotherapy, chemo-free treatment using anti-PD-1 antibodies combined with lenvatinib may be an effective treatment for patients with unresectable GBC, especially those who are not suitable for systemic chemotherapy.

## 2. Patients and Methods

### 2.1. Ethics Statement

The present study was approved by the Ethics Committee of Peking University International Hospital and adhered to the ethical principles of both institutional and national research committees. Moreover, it was aligned with the 1964 Declaration of Helsinki, including its later amendments and analogous ethical standards. Given the retrospective nature of this study, which involved analysis of previously collected clinical data, the requirement for written informed consent was waived.

### 2.2. Study Design and Patients

A total of 64 patients with histologically confirmed stage IV GBC were retrospectively analyzed. Patients were divided into two groups: a chemotherapy group and a combined therapy group. Patients in the chemotherapy group received the GC regimen, and those in the combined therapy group received an anti-PD-1 antibody combined with lenvatinib (Camrelizumab + Lenvatinib) at Peking University International Hospital from March 2018 to October 2022. Enrolled patients met the following eligibility criteria: (1) Histologically proven stage IV GBC, using the EnVision two-step method. EnVision is a very sensitive detection method for routine IHC, firstly reported in 1998 [21]. Morphological characteristics and expression of CK7, CK20, CK19, and mucoprotein were the central criteria for adenocarcinoma. (2) A follow-up of at least eight weeks. (3) Those who had not previously received any anti-tumor treatment. Two patients in the chemotherapy group and two patients in the combination therapy group were followed up less than eight weeks. One patient in the combination therapy group accepted previous systemic chemotherapy. The five patients were excluded from the cohort.

### 2.3. Data Collection and Clinical Outcome Assessment

The data on patients’ clinicopathological characteristics and course of treatment were independently collected and organized by two researchers, and the imaging data were independently assessed by two radiologists. Patients were followed up with every 8–12 weeks. The clinical objective responses were measured and evaluated by radiologists and oncologists from Peking University International Hospital based on the Response Evaluation Criteria in Solid Tumors (RECIST) v1.1 [22] and defined as complete response (CR), partial response (PR), stable disease (SD), or progressive disease (PD). The progression-free survival (PFS), OS, objective response rate (ORR) [22], and disease control rate (DCR) were determined to evaluate clinical efficacy. PFS was defined as the period from the time of treatment to disease progression or patient death due to any cause. OS was defined as the period from the time of treatment to patient death due to any cause or the last day of follow-up. ORR was defined as the proportion of patients with a complete response or partial response to treatment according to RECIST v1.1. DCR was defined as the percentage of patients with advanced cancer whose therapeutic intervention has led to a complete response, partial response, or stable disease. Adverse events (AEs) were assessed according to the Common Terminology Criteria for Adverse Events version 4.0 [23].

### 2.4. Treatment

From March 2018 to October 2022, patients in the chemotherapy group received the GC regimen as the first-line treatment according to NCCN guidelines for hepatobiliary cancers and the Chinese Society of Clinical Oncology Guidelines for Diagnosis and Treatment of Biliary Tract Malignancies. Patients not suitable for or unwilling to undergo conventional chemotherapy were suggested with anti-PD-1 antibody + lenvatinib as the first-line treatment and enrolled in the combined therapy group in this study. Informed consent was signed by the patient or the representative before treatment. Immunohistochemical staining of PD-L1 in biopsy tissue was performed before treatment. The expression level of PD-L1 was quantified using the tumor proportion score (TPS). Not all patients in the combined therapy group accepted the genetic test, mainly because of its cost; thus, data on tumor mutation burden (TMB) and microsatellite instability (MSI) were not included in this study.

Treatment in the chemotherapy group included sequential intravenous infusion of gemcitabine with a fixed dose of 1000 mg/m^2^ and cisplatin with a fixed dose of 25 mg/m^2^ on days 1 and 8, which was repeated every 3 weeks. Based on patients’ tolerance, the systemic chemotherapy lasted for a maximum of 8 cycles, followed by gemcitabine monotherapy. Treatment in the combined therapy group included camrelizumab (first anti-PD-1 antibody available in our hospital) at a fixed dose of 200 mg or 3 mg/kg (body weight) every 3 weeks, and lenvatinib was concomitantly administered orally once a day at a dose of 8 mg. The date of treatment, drug dosage, radiological evaluation, laboratory data, and AEs were recorded.

### 2.5. Statistical Analysis

The baseline characteristics and response data of the two groups were compared using the chi-squared test or Fisher’s exact test for categorical variables. Normally distributed data were compared using the Student’s *t*-test, whereas the Mann–Whitney U test was used for skew data. Survival analysis was performed using Kaplan–Meier curves, with a *p*-value determined using the Breslow test. Hazard ratios (HRs) were estimated using the Cox proportional hazards regression analysis. A two-sided *p*-value of <0.05 was considered statistically significant. The last follow-up was on 30 March 2023. All statistical analyses were performed using SPSS (version 25.0; SPSS, Chicago, IL, USA).

## 3. Results

### 3.1. Patient Characteristics

A total of 64 patients with advanced GBC who were treated with combined therapy (anti-PD-1 antibody + lenvatinib) or chemotherapy (GC regimen) as first-line therapy were recruited. Demographic and baseline characteristics are summarized in Table 1. All patients (100.00%) had an Eastern Cooperative Oncology Group (ECOG) performance status of 0–1, and 57 (89.06%) patients presented with good liver function and Child–Pugh grade A. The serum bilirubin levels were similar in both groups (all *p* < 0.05). Three patients accepted biliary drainage before drug treatment in the combined therapy group, while no cases of biliary drainage occurred in the chemotherapy group. Adenocarcinoma was the most frequent histopathological subtype of GBC, occurring in all 64 (100.00%) patients. Thirty-four (53.13%) patients were poorly differentiated. All patients had metastatic lesions in the liver (64/64, 100.00%) and lymph nodes (64/64, 100.00%). Pulmonary metastasis occurred in 5 patients (7.81%) and peritoneal metastasis was observed in 18 patients (28.13%). In terms of the Tumor Node Metastasis Classification (TNM) stage, all patients were diagnosed with stage IV GBC, and none had previously undergone any anti-tumor treatment.

### 3.2. Efficacy

All patients were followed up for at least 3 months, with a median follow-up time of 10.00 months (IQR, 6.25–14.00 months). The median treatment duration was 8.00 months (IQR, 5.00–11.00 months) in the combined therapy group and 6.00 months (IQR, 3.50–7.50 months) in the chemotherapy group. The median treatment cycle was 10 (IQR, 6–14) in the combined therapy group and 6 (IQR, 4–9) in the chemotherapy group.

**Figure 1:**
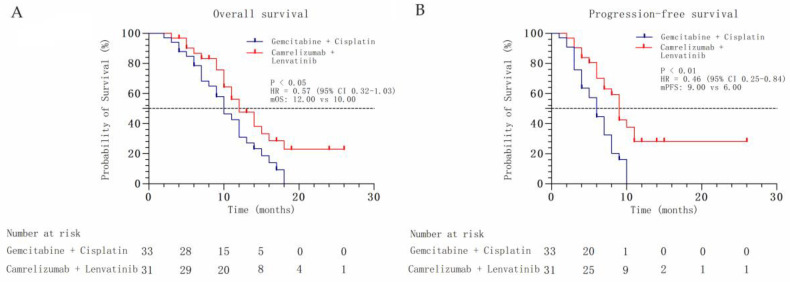
(**A**) Kaplan–Meier analysis of overall survival between the chemotherapy and combined therapy groups. (**B**) Kaplan–Meier analysis of progression-free survival between the chemotherapy and combined therapy groups.

**Figure 2:**
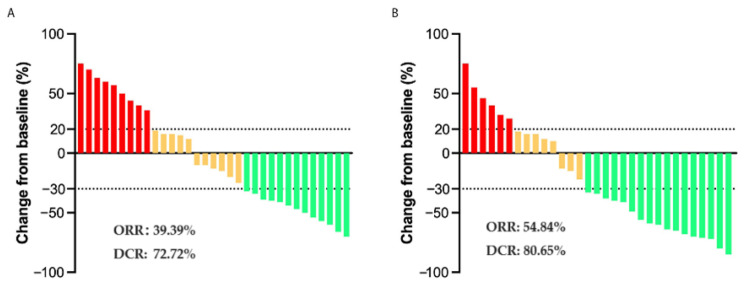
(**A**) Maximum percentage change in the sum of the diameters of the target lesions from baseline in chemotherapy group. (**B**) Maximum percentage change in the sum of the diameters of the target lesions from baseline in combined therapy group. Red bars represent PD, yellow bars represent SD and green bars represent PR.

**Figure 3:**
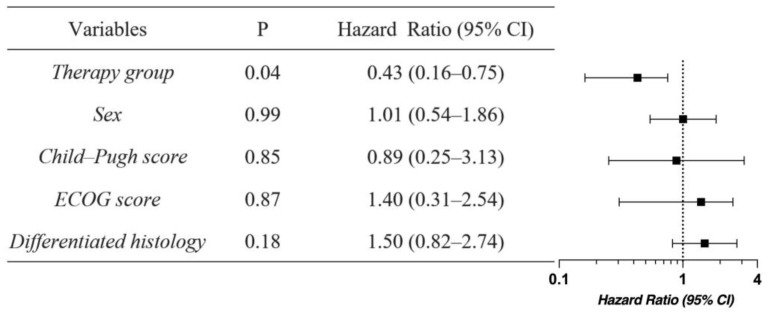
Subgroup analyses of pre-specified baseline factors.

Forty-five (70.31%) deaths were recorded. The median OS was 12.00 months (95% confidence interval (CI): 8.80–15.20 months) for the combined therapy group and 10.00 months (95% CI: 7.63–12.37 months) for the chemotherapy group (hazard ratio (HR), 0.57; 95% CI: 0.32–1.03; *p* < 0.05, Figure 1A). The median PFS was 9.00 months (95% CI: 7.88–10.12 months) for the combined therapy group and 6.00 months (95% CI: 4.65–7.35 months) for the chemotherapy group (HR, 0.46; 95% CI: 0.25–0.84; *p* < 0.01, Figure 1B). The ORR was higher in the combined therapy group than in the chemotherapy group (54.84 vs. 39.39%, *p* = 0.22), and a similar tendency was observed in terms of DCR (80.65 vs. 72.72%, *p* = 0.46), although the difference was not statistically significant (Figure 2, Table 2). HRs for death were analyzed according to pre-specified baseline factors. The HR for the combined therapy group was 0.43, indicating that combined therapy contributed to patient survival (*p* = 0.04, Figure 3).

**Figure 4:**
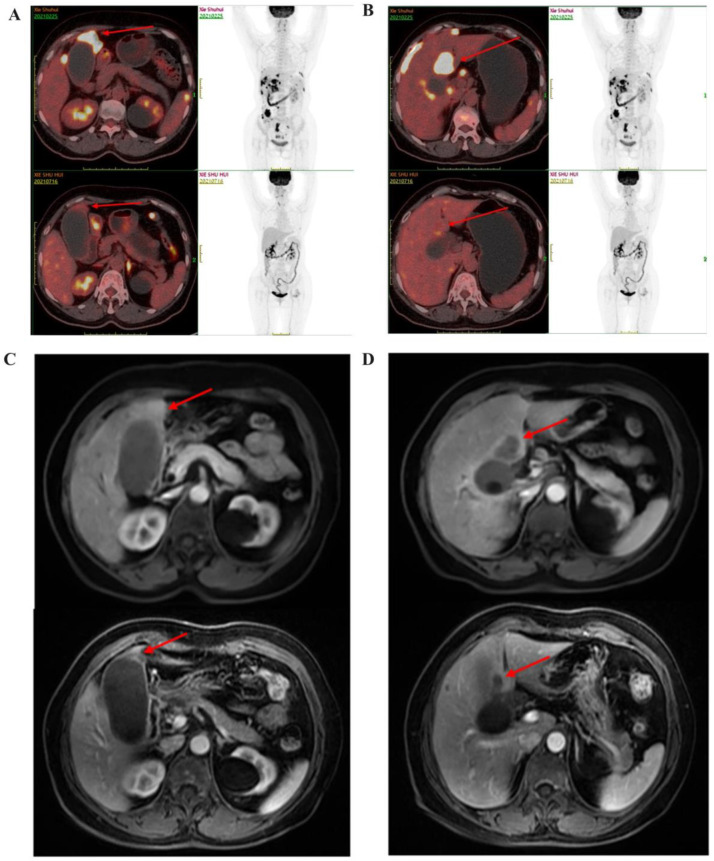
Clinical efficacy of a case in combined treatment group upon radiological evaluation. (**A**,**B**) Positron emission tomography/computed tomography (PET/CT). (**A**) Glucose metabolism of the mass at the bottom of the gallbladder was decreased (red arrow). (**B**) Glucose metabolism in the liver parenchyma around the gallbladder was decreased (red arrow). The upper images (**A**,**B**) are the baseline images in February 2021, and the lower images (**A**,**B**) are the follow-up images in July 2021. (**C**) Enhanced magnetic resonance imaging (MRI) revealed the shrinkage of the mass at the bottom of the gallbladder (red arrow). (**D**) Enhanced MRI revealed the shrinkage of the mass in the liver parenchyma around the gallbladder (red arrow). The upper images (**C**,**D**) are the baseline images in February 2021, and the lower images (**C**,**D**) are the follow-up images in October 2021.

At the end of the follow-up period, 19 of 64 GBC patients were still alive. Among them, one patient diagnosed with GBC, with intrahepatic, lymph node, and peritoneal metastases, was treated with an anti-PD-1 antibody combined with lenvatinib [24]. The TPS of PD-L1 expression was 30%. The patient underwent surgical resection 8 months after combined therapy and the stage of disease was downgraded from IVb to IIIb (Figure 4). After postoperative pathological examination, the pCR was achieved. The patient resumed combined therapy 2 weeks after radical resection, and no recurrence was found during follow-up.

### 3.3. Safety

AEs observed across all grades are summarized in Table 3. A combination of anti-PD-1 antibody and lenvatinib was well-tolerated by the patients. The most frequently reported treatment-related AEs in the chemotherapy group were fatigue (23/33, 69.70%), with a median of 2.00 cycles of treatment (IQR, 1.00–2.00 cycles), and leukopenia (15/33, 45.45%), with a median of 3.00 cycles of treatment (IQR, 2.00–3.00 cycles). Grades 3–4 treatment-related AEs were reported in 4 (12.12%) patients, including leukopenia at 5 cycles of treatment, vomiting at 6 cycles of treatment, and diarrhea at 5 cycles of treatment. The most frequently reported treatment-related AEs in the combined therapy group were fatigue (12/31, 38.71%) with a median of 2.00 cycles of treatment (IQR, 1.25–2.75 cycles) and decreased appetite (6/31, 19.35%) with a median of 1.50 cycles of treatment (IQR, 1.00–2.25 cycles). Grades 3–4 treatment-related AEs were reported in 3 (9.68%) patients, including hypertension at 9 cycles of treatment, diarrhea at 12 cycles of treatment, and gastrointestinal hemorrhage at 12 cycles of treatment. Gastrointestinal hemorrhage was caused by tumor invasion of the duodenum and was not a true treatment-related AEs. Regarding hematologic toxic effects, no patient experienced AEs in the combined therapy group compared with the chemotherapy group, demonstrating the advantage of the combined therapy. Disease progression was the main reason for treatment discontinuation.

## 4. Discussion

GBC is one of the most common malignant tumors of the biliary tract, with the sixth highest incidence among malignant tumors of the digestive system [25]. With a high degree of malignancy, the majority of GBC patients are in an advanced stage at the time of diagnosis and are thus unable to undergo radical resection. Moreover, most patients have an overall poorer prognosis. Therefore, systemic therapies are particularly important in the treatment of GBC. According to two high-quality clinical studies, ABC-02 [26] and BT22 [27], the GC regimen has become the standard first-line chemotherapy, extending the OS to 11.7 months. However, it has been reported recently that the effectiveness of chemotherapy drugs such as cisplatin, gemcitabine, and 5-fluorouracil is unsatisfactory for patients with GBC, with a response rate of 35.5% and an OS of 7.2 months [28]. The rapid development of targeted therapy and immunotherapy offers new prospects for the treatment of patients with advanced GBC [29]. Targeted therapy and immunotherapy may be effective approaches to improve the prognosis of advanced tumors and provide patients with the opportunity for radical surgery.

This study retrospectively analyzed 64 patients with advanced GBC who received systemic therapy to evaluate the efficacy and safety of anti-PD-1 antibody combined with lenvatinib as first-line therapy. Chemotherapy is the primary treatment for advanced biliary tract cancer (BTC) [30]. Our study showed that the median PFS and OS of patients receiving the GC regimen were lower than those reported in the clinical trial ABC-02 [26]. The ORR was 39.39%, which was higher than that previously reported [28]. These may be due to the heterogeneity of GBC and the small sample size of our cohort. To some extent, the effectiveness of the GC regimen may be unsatisfying for unresectable stage IV GBC. GBC should be considered a special type of BTC and treated differently.

In recent years, ICIs have become a research hotspot in immunotherapy, and anti-PD-1 antibodies have been successfully used for the treatment of various tumors [14,31]. However, few clinical reports have considered ICI treatment for GBC. The efficacy of the anti-PD-1 antibody against BTC has been validated in two clinical trials, KEYNOTE-028 and KEYNOTE-158. The ORR of the anti-PD-1 antibody (pembrolizumab) in patients with advanced BTC was 13 and 5.8%, and the median OS was 5.7 and 7.4 months for KEYNOTE-028 and KEYNOTE-158 trials, respectively [32]. Sun et al. [33] compared the efficacy of anti-PD-1 antibody combined with chemotherapy and anti-PD-1 antibody or chemotherapy alone and found that the prognosis of the combination therapy was significantly better than that of monotherapy. Another study showed that nivolumab + GC regimens achieved a PR of 37% in patients with BTC (containing 33% GBC), which was higher than the PR of the nivolumab or GC regimen alone [34]. However, although a combined therapy of immunotherapy with chemotherapy significantly improved PFS and OS, AEs and poor tolerance compromised the overall therapeutic effectiveness and the patient’s quality of life.

Targeted therapy has strong specificity and relatively few side effects and has played a huge role in the treatment of various solid tumors. Lenvatinib is a multi-target TKI that targets the vascular endothelial growth factor receptor 1 (VEGFR-1), VEGFR-2, and VEGFR-3, inhibiting angiogenesis in tumor tissues. Studies have shown that lenvatinib has curative effects on multiple solid tumors [17,35,36]. Furthermore, the anti-PD-1 antibody has been reported to enhance the efficacy of lenvatinib by altering the immune system, and lenvatinib can enhance the anti-tumor efficacy of anti-PD-1 immunotherapy [37]. Anti-PD-1 antibody combined with lenvatinib has shown synergistic therapeutic effects on advanced liver cancer [38]. Our analysis showed that patients in the combined therapy group had a significantly longer median PFS and OS than those in the chemotherapy group, suggesting the efficacy of the combination therapy. Considering the retrospective nature and limited sample size of our cohort, the benefit of the combined therapy to patient survival is worth further investigation. The ORR was 54.84 and 39.39% in combined therapy and chemotherapy groups, respectively, although the difference was not significant. A similar trend was observed in DCR between the two groups. This may have been due to the limited sample size. The advantages of anti-PD-1 antibody plus lenvatinib in ORR and DCR require further clinical trials with larger sample sizes.

In addition to efficacy, safety and tolerance are important considerations for the selection of therapeutic agents. Our study found that combination therapy had a manageable and well-tolerated safety profile. Few patients reported treatment-related AEs of grades 3–4, and no patients discontinued treatment due to treatment-related adverse reactions. The incidence of grade 3–4 AEs was lower in the combined therapy group than in the chemotherapy group. Fatigue and decreased appetite were the most common treatment-related AEs in the combined therapy group, whereas hematologic toxicity was the most frequent treatment-related AE in the chemotherapy group, which was also reported in the ABC-02 randomized phase III trial [22]. None of the patients in the combined therapy group experienced hematological toxicity, which underscores the advantages of the combined therapy.

PD-L1 immunohistochemical staining was conducted in all patients in the combined therapy group. It was previously reported that the ORR and PFS of advanced BTC patients with positive PD-L1 expression were superior to those of PD-L1 negative patients after treatment with lenvatinib combined with PD1 monoclonal antibody [21]. In our cohort, the TPS of the successful conversion case was 30%. The importance of PD-L1 expression to the clinical outcome of the combined therapy was beyond the scope of this study. However, our previous report found that the expression of tumor PD-L1 in patients with objective response was significantly higher than that in patients with disease progression [24]. Thus, PD-L1 may be a promising indicator for predicting response, which certainly requires further exploration. Like PD-L1 expression, TMB and MSI could potentially impact treatment outcomes of combined therapy; however, a genetic test is not widely accepted among patients, owing to its high cost. Thus, TMB and MSI data were incomplete in this study. Future clinical trials should endeavor to overcome this challenge.

GBCs can cause biliary obstruction, mainly because of infiltration to the biliary tree. Three patients in the combined therapy group were complicated with jaundice and not suitable for the combined therapy. Biliary drainage relieved jaundice and facilitate systemic treatment. No liver dysfunction was identified in this group during treatment. It was reported that preoperative biliary drainage of jaundiced GBC patients decreased the risk of postoperative liver failure; however, it did not impact long-term outcomes [39]. In our study, it might be hypothesized that biliary drainage decreased the risk of liver dysfunction caused by systemic treatment. To this point, future investigations are necessary.

Currently, clinical data on targeted therapy combined with immunotherapy for GBC are scanty. A prospective single-arm study reported the efficacy of anti-PD-1 antibody plus lenvatinib as a non-first-line treatment for BTC patients, including 32 BTC patients enrolled (6 GBC patients). All the patients suffered disease progression in response to systemic chemotherapy, and the results showed that the median OS was 11.0 months [40]. Zuo et al. [20] investigated an anti-PD-1 antibody combined with lenvatinib in 31 patients with advanced GBC. The baseline data before treatment were heterogeneous: all patients were in stage III or IV, and 24 patients (77.4%) received related treatments before anti-PD-1 antibody combined with lenvatinib therapy, including surgery, systemic chemotherapy, and radiotherapy. Our study enrolled more homogenous patients without any prior anti-tumor therapy and mainly focused on the efficacy of the PD-1 monoclonal antibody inhibitor plus lenvatinib in stage IV GBC.

To the best of our knowledge, there is no study comparing combined therapy with conventional chemotherapy as first-line treatment in advanced GBC. The present study provides valuable data concerning biliary tract cancer. Nonetheless, this study has some limitations. Firstly, the sample size is limited; hence, future studies should be conducted with larger sample sizes to reduce bias. Secondly, this study was a retrospective analysis, with a low grade of evidence. Thus, prospective randomized controlled trials are needed to validate our findings.

## 5. Conclusions

In summary, this study demonstrated that anti-PD-1 antibody combined with lenvatinib improved the median PFS and OS and is a promising therapeutic modality for advanced GBC. We believe that a rational selection of patients who are sensitive to immunotherapy and the exploration of optimized immunotherapy combined with targeted therapy will become a hot research topic in the future. This study was a single-center retrospective clinical study with several limitations. Therefore, well-designed multicenter and prospective studies are required to further validate the effectiveness of anti-PD-1 antibody combined with lenvatinib in the treatment of advanced GBC.

## Figures and Tables

**Table 1:** Baseline characteristics.

Characteristic	Gemcitabine + Cisplatin (N = 33)	Camrelizumab + Lenvatinib (N = 31)	*p*
Age, (median, IQR)	62 (56–66)	67 (61–70)	0.06
Sex, n (%)			0.79
Male	16 (48.48)	14 (45.16)	
Female	17 (51.52)	17 (54.84)	
Child–Pugh score, n (%)			0.48
A	28 (84.85)	29 (93.55)	
B	5 (15.15)	2 (6.45)	
ECOG performance status, n (%)			0.78
0, n (%)	28 (84.85)	28 (90.32)	
1, n (%)	5 (15.15)	3 (9.68)	
CA19-9, U/mL (median, IQR)	45.00 (16.20–166.45)	40.00 (11.90–220.30)	0.67
<200, n (%)	25 (75.76)	22 (70.97)	
≥200, n (%)	8 (24.24)	9 (29.03)	
CEA, U/mL (median, IQR)	2.70 (1.80–6.00)	2.90 (2.00–6.10)	0.93
Serum TBIL	17.90 (13.10–21.75)	16.90 (12.90–20.60)	0.62
Serum DBIL	4.70 (3.25–6.25)	5.0 0(3.60–7.80)	0.17
Biliary Drainage, n	0	3	
Histology, n (%)			
Adenocarcinoma	33 (100.00)	31 (100.00)	
Differentiated histology, n (%)			0.25
Moderately differentiated	18 (54.55)	13 (41.94)	
Poorly differentiated	15 (45.45)	16 (51.61)	
Undifferentiated	0	2 (6.45)	
Previous therapy, n (%)			
None	33 (100.00)	31 (100.00)	
Metastatic site, n (%)			
Intrahepatic	33 (100.00)	31 (100.00)	
Lymph nodes	33 (100.00)	31 (100.00)	
Peritoneum	12 (36.36)	6 (19.35)	
Lung	2 (6.06)	3 (9.68)	
TNM stage, n (%)			
IV	33 (100.00)	31 (100.00)	
PD-L1 expression TPS (%) (median, IQR)	NA	9.40 (5.00–30.20)	
Frist antitumor therapy, n (%)			
Gemcitabine + Cisplatin	33 (100.00)	0	
Camrelizumab + Lenvatinib	0	31 (100.00)	
Subsequent antitumor therapy, n (%)			
Radical surgery resection	0	1 (3.23)	
Systemic chemotherapy	4 (12.12)	3 (9.68)	
Radiotherapy	2 (6.06)	1 (3.22)	
Immunotherapy and targeted therapy	3 (9.09)	0	
Type of anti-PD-1 antibody, n (%)			
Camrelizumab	0	31 (100.00)	

ECOG, Eastern Cooperative Oncology Group; CA19-9, carbohydrate antigen 19-9; CEA, carcinoembryonic antigen; TBIL, total bilirubin; DBIL, direct bilirubin; TNM, tumor node metastasis classification.

**Table 2:** Tumor response to treatment for the overall cohort.

Therapeutic Response Assessment	Gemcitabine + Cisplatin (N = 33)	Camrelizumab + Lenvatinib (N = 31)	*p*
Objective response rate, (ORR, n, %)	13 (39.39)	17 (54.84)	0.22
Disease control rate, (DCR, n, %)	24 (72.73)	25 (80.65)	0.46
Complete response (CR, n, %)	0	0	
Partial response (PR, n, %)	13 (39.39)	17 (54.84)	
Stable disease (SD, n, %)	11 (33.33)	8 (25.80)	
Progressive disease (PD, n, %)	9 (27.27)	6 (19.35)	

ORR, objective response rate; DCR, disease control rate; CR, complete response; PR, partial response; SD, stable disease; PD, progressive disease.

**Table 3:** Treatment-related adverse events.

Adverse Events (AEs)	Gemcitabine + Cisplatin (N = 33)		Camrelizumab + Lenvatinib (N = 31)	
	Grade 1–4, n (%)	Grade 3–4, n (%)	Grade 1–4, n (%)	Grade 3–4, n (%)
Hematologic toxic effects				
Leukopenia	15 (45.45)	1 (3.03)	0	0
Anemia	6 (18.18)	0	0	0
Thrombocytopenia	6 (18.18)	0	0	0
Nonhematologic toxic effects				
Fatigue	23 (69.70)	0	12 (38.71)	0
Hypertension	3 (9.09)	0	2 (6.45)	1 (3.23)
ALT or AST elevation	6 (18.18)	0	2 (6.45)	0
Decreased appetite	20 (60.61)	0	6 (19.35)	0
Abdominal pain	0	0	3 (9.68)	0
Vomiting	10 (30.30)	2 (6.06)	2 (6.45)	0
Diarrhea	3 (9.09)	1 (3.03)	3 (9.68)	1 (3.23)
Decreased weight	14 (42.42)	0	2 (6.45)	0
RCCEP	0	0	1 (3.23)	0
Gastrointestinal hemorrhage	0	0	1 (3.23)	1 (3.23)

ALT, alanine aminotransferase; AST, aspartate aminotransferase; RCCEP, reactive cutaneous capillary endothelial proliferation.

## Data Availability

The datasets generated in the current study are available from the corresponding author upon reasonable request.
